# Hemodynamic responses to 1 MAC desflurane inhalation during anesthesia induction with propofol bolus and remifentanil continuous infusion: a prospective randomized single-blind clinical investigation

**DOI:** 10.1186/s12871-023-02002-6

**Published:** 2023-02-22

**Authors:** Jong Wha Lee, Mi Kyeong Kim, Ji Young Kim

**Affiliations:** 1 Department of Anesthesiology and Pain Medicine, Ewha Womans University School of Medicine, Seoul, Republic of Korea; 2 Department of Anesthesiology and Pain Medicine, Kyung Hee University Medical Center, Seoul, Republic of Korea; 3 Department of Anesthesiology and Pain Medicine, Anesthesia and Pain Research Institute, Yonsei University College of Medicine, Seoul, Republic of Korea; 4 Department of Anesthesiology and Pain Medicine, Gangnam Severance Hospital, Yonsei University Health System, 211, Eonju-Ro, Gangnam-Gu, Seoul, 06273 Republic of Korea

**Keywords:** Desflurane, Sevoflurane, Remifentanil, Hemodynamic response, Age

## Abstract

**Background:**

Desflurane is not recommended during anesthesia induction because of its sympathetic stimulation effect, particularly in patients with myocardial ischemic disease. To date, the hemodynamic response to 1 MAC desflurane inhalation in combination with remifentanil infusion during anesthesia induction has rarely been reported.

**Methods:**

This investigation was designed to compare hemodynamic responses to 1 MAC desflurane (group D, *n* = 200) with sevoflurane (group S, *n* = 200) during anesthesia induction and endotracheal intubation in adult patients undergoing elective spine surgery. Subgroup analysis of the different age subgroups was also performed. With continuous infusion of remifentanil 0.1 μg/kg/min, anesthesia was induced with propofol bolus, and endotracheal intubation was performed after muscle relaxation. Heart rate (HR) and mean arterial blood pressure (MAP) were measured every minute for 5 min after anesthesia induction (T1-5) and after endotracheal intubation (T6-10).

**Results:**

HR was significantly higher in group D (*n* = 182) than in group S (*n* = 173) at T3-10 except at T6 (1 min after intubation) (all *P* < 0.05). In the age-based subgroup analyses, which subdivided the group D and S into four subgroups based on patient’s age, the changes in HR from baseline values were significantly different between the coeval subgroups of patients in their 20–29 years and 30–39 years of age (all *P* < 0.05). MAP was reduced from baseline value, irrespective of group and age.

**Conclusion:**

Inhalation of 1 MAC desflurane during anesthesia induction with propofol bolus and remifentanil continuous infusion and during endotracheal intubation was more likely to induce elevations in HR more likely than 1 MAC sevoflurane, especially in younger patients.

**Trial registration:**

This study was registered in the Clinical Research Information Service (CRIS, http://cris.nih.go.kr) of the Republic of Korea on Feb 12, 2016 (Registration No. KCT 0,001,813).

**Supplementary Information:**

The online version contains supplementary material available at 10.1186/s12871-023-02002-6.

## Background

Since desflurane had been introduced into clinical anesthetic practice in the 1990’s, it was frequently reported that desflurane had sympathetic stimulation effect. Tachycardia and/or hypertension was observed when desflurane of > 1 MAC was rapidly administered during anesthesia induction and steady-state anesthesia [1–4]. However, recent investigations using spectral analysis of heart rate (HR) variability have found that desflurane-anesthetized animals exhibit increases in HR due to parasympathetic inhibition [5, 6]. A preliminarily study also reported parasympathetic inhibition in patients [7]. These detrimental hemodynamic responses to desflurane have prevented its use in patients at risk of myocardial infarction, including hypertension or ischemic heart disease [8].

Inhalation agent is not employed in concentrations as high as it once was, particularly with recently introduced potent, short-acting opioid, remifentanil. But inhalation agent is still co-administered at moderate doses with various induction agents to maintain hemodynamic stability during anesthesia induction and endotracheal intubation. To date, the hemodynamic response to 1 MAC desflurane inhalation in combination with remifentanil infusion during anesthesia induction has rarely been reported. In addition, the sympathetic stimulation effect of desflurane has previously demonstrated in young and healthy volunteers [1–4]. There are no reports on whether the hemodynamic response to desflurane inhalation during anesthesia induction is age-dependent.

The primary aim of this investigation was to compare hemodynamic responses to 1 MAC desflurane with those to 1 MAC sevoflurane during anesthesia induction with propofol bolus and remifentanil continuous infusion in adult patients undergoing elective spine surgery. The secondary aim was to compare the hemodynamic responses of patients in different age subgroups. The hypothesis was that the sympathetic stimulation effect would not be observed when administering a low concentration desflurane in combination with remifentanil infusion.

## Methods

After obtaining approval from the local ethics committee (4–2015-0683) on 14/09/2015 and written informed consent from participants, patients undergoing elective spine surgery were enrolled in this prospective, randomized, single-blinded trial. This study was registered in the Clinical Research Information Service (CRIS, http://cris.nih.go.kr) of the Republic of Korea (KCT 0,001,813) on 12/02/2016.

### Patients

Patients 20–59 years old undergoing elective spine surgery were enrolled. Patients with a history of uncontrolled or untreated hypertension and/or diabetes with neuromuscular complications, active upper respiratory infection, asthma, cancer metastasis or traumatic injury in spine, cerebrovascular accident, renal insufficiency, valvular or ischemic heart disease or metabolic disorders were excluded. Additionally, patients with a history of difficult airway or morphological characteristics predicting difficult airway (i.e., hypoplastic mandible, Mallampati class III or higher, and thyromental distance of < 2 fingerbreadths) were also excluded.

### Interventions

Four hundred patients were randomized to receive either 1 MAC desflurane (group D, *n* = 200) or sevoflurane (group S, *n* = 200) using a computer-generated random number in a sealed envelope. The vol% of 1 MAC desflurane or sevoflurane was calculated using an age-based formula suggested by Mapleson WW [9]. The formula was as follows: $\mathrm{MAC}={\mathrm{MAC}}_{40} \times {10}^{b(age-40)}$, where MAC_40_ = MAC at age of 40 years, 6.6% and 1.8% for desflurane and sevoflurane, respectively, b = -0.00269, and age = patient’s age in years (> 1). The end-tidal fraction (F_ET_) of the designated inhalation agent was monitored to maintain the F_ET_ within 70–80% of the inspiratory fraction (F_i_) of the designated agent.

### Anesthetic management

No premedication was administered. Upon arrival in the OR, routine monitoring devices, including ECG lead II, pulse oximetry, non-invasive blood pressure and bispectral index, were applied. After confirming the patency of the peripheral intravenous (IV) route, remifentanil 0.1 μg/kg/min was initiated along with the rapid infusion of IV fluid. Thereafter, no further manipulation of the patient occurred in order to avoid overstressing the patient for 5–10 min. The hemodynamic parameters, including HR and mean arterial blood pressure (MAP), were measured twice at an interval of 3 min. The average value of two measurements was used as baseline value for hemodynamic comparison (T0).

After the IV injection of glycopyrrolate 0.2 mg, anesthesia was induced with propofol of 1–1.5 mg/kg IV over 30 s. If the patient was able to maintain a verbal response, propofol 10 mg IV was administered every 10 s. When loss of consciousness was confirmed, 1 MAC desflurane or sevoflurane in oxygen/air with a flow rate of > 5 L/min was administered via a face mask. To facilitate endotracheal intubation, rocuronium 0.6 mg/kg IV was administered along with lidocaine 30–40 mg IV to prevent injection pain. An end-tidal CO_2_ (E_T_CO_2_) level of 35–40 mmHg and a peak airway pressure of < 25 cm H_2_O were maintained with manual ventilation for 5 min. The patient’s tracheal was intubated using direct laryngoscopy or light wand, depending on the need for lumbar/thoracic or cervical spine surgery, respectively. Appropriate placement of the endotracheal tube was confirmed using bilateral chest auscultation and waveform observations of E_T_CO_2_. The ventilator was set to maintain E_T_CO_2_ between 35 and 40 mmHg with a tidal volume of 8 ml/kg.

### Measurements

The HR and MAP were recorded every minute for 5 min after anesthesia induction (T1-5) and for 5 min after endotracheal intubation (T6-10). When airway maintenance or endotracheal intubation was difficult, requiring oral or nasal airway insertion or > 3 attempts for intubation, the patient was excluded from the analysis.

### Rescue medications

In case of hypertension or tachycardia, defined as an increase in HR or MAP by > 30% of baseline value, a bolus dose of remifentanil 0.5 μg/kg was administered as the initial rescue medication. If hypertension or tachycardia persisted for > 1 min, IV esmolol 5–10 mg was administered as the secondary rescue medication. Hypotension, defined as a decrease in MAP by > 30% of baseline value, was treated before intubation as follows. Initially, the remifentanil infusion dose was reduced by half. If hypotension persisted for > 1 min, the inhalation anesthetic concentration was reduced by half. In case of persistent hypotension, endotracheal intubation was performed immediately. When the MAP was reduced by > 30% of baseline value after endotracheal intubation, IV ephedrine 4 mg was given. If hypotension persisted further, IV phenylephrine 100 μg was administered as the secondary rescue medication until the MAP was maintained at > 70% of baseline value. Bradycardia, defined as a decrease in HR by > 30% of baseline value, was treated with IV atropine 0.5 mg. The frequency of rescue medication administration during anesthesia induction and endotracheal intubation was recorded.

### Outcome variables

The primary endpoint of this study was to compare the hemodynamic responses (HR and MAP) to 1 MAC desflurane with those to 1 MAC sevoflurane during the anesthesia induction and endotracheal intubation. The secondary endpoint was to evaluate the age-dependent differences in hemodynamic responses. To evaluate the age-dependent difference, groups D and S were subdivided into four subgroups based on patient’s age: 20–29 years, 30–39 years, 40–49 years, and 50–59 years old (D20, D30, D40, and D50 for group D; S20, S30, S40, and S50 for group S).

### Sample size and statistical analysis

To obtain a sample power of 0.8, it was calculated to have at least 45 patients for each age subgroup on the assumption of a medium effect size of 0.25 and α of 0.05, using G*Power software (Ver. 3.1.9.2, Universität Kiel, Germany, http://www.gpower.hhu.de/). Adjusting for 10% drop-out, it was decided to recruit 50 patients for each age subgroup, resulting in a total of 400 patients in groups D and S.

The specific characteristics of the recruited patients were statistically evaluated as follows. Discrete data are shown as a number (percentage) of patients and compared using the χ^2^ test or Fisher’s exact test, and continuous variables are represented as the mean ± standard deviation and statistically evaluated using independent t-test between the two groups.

For statistical analyses of repeatedly measured data, a linear mixed-effect model (LMM) was adopted after the normality test. Group, time, and group × time were considered fixed effects and time was clustered within the patients. Post hoc tests comparing to baseline value were performed with Bonferroni correction. Statistical analyses were performed using SAS (version 9.4, SAS Inc., Cary, NC, USA). A *P* < 0.05 was employed as statistically significant.

## Results

### Characteristics

**Fig. 1:**
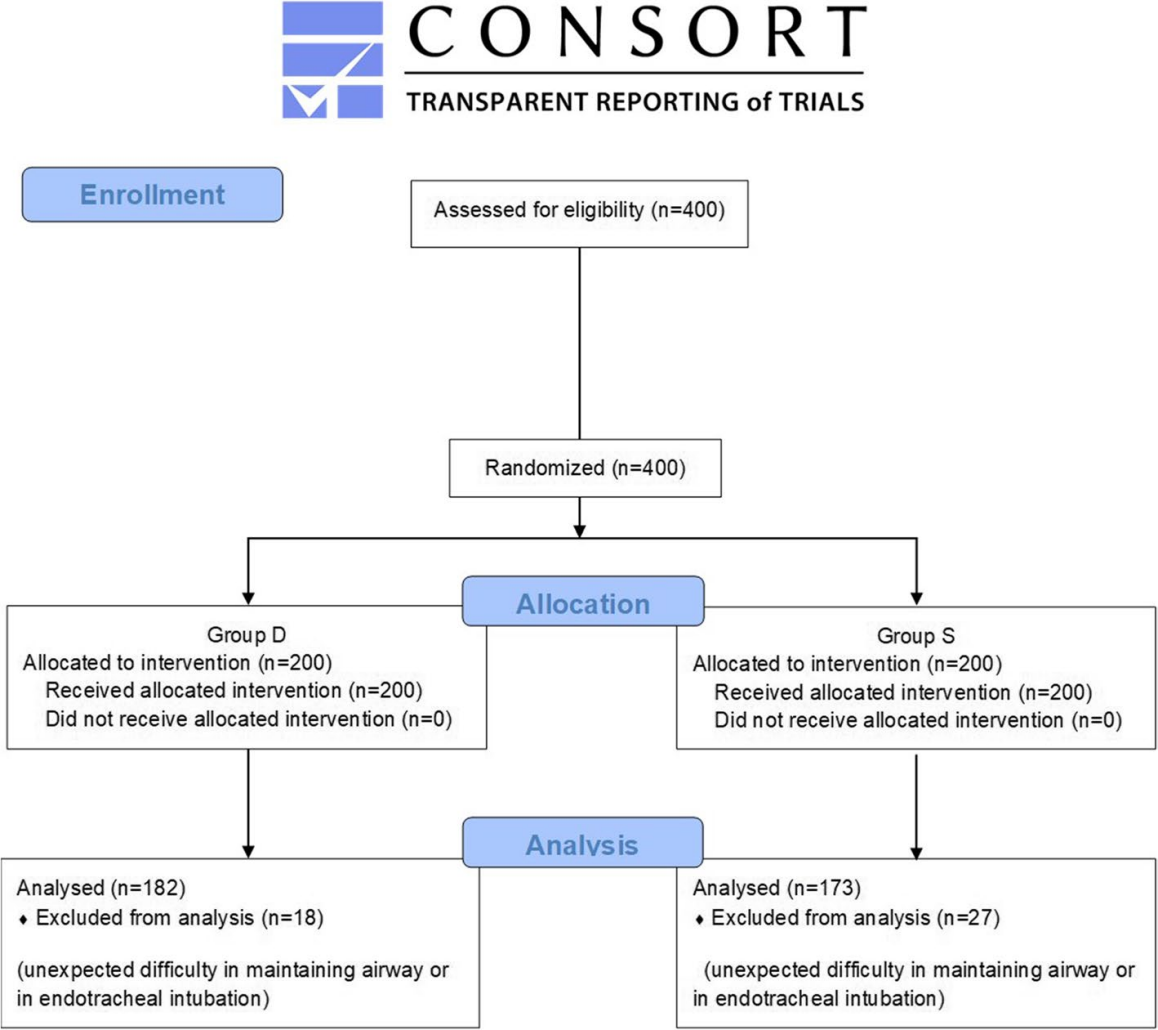
CONSORT flow diagram, showing cases included and reasons for exclusion. CONSORT, Consolidated Standards of Reporting Trials

A total of 400 patients undergoing elective spine surgery were enrolled and randomly allocated into group D or S; however, 45 patients (18 in group D and 27 in group S) were excluded from the final analyses due to unexpected difficulties in airway maintenance or endotracheal intubation. The remaining 355 patients (182 in group D, and 173 in group S) were included in the statistical analyses (Fig. 1). Patient characteristics, including co-morbidities and data on induction of anesthesia and endotracheal intubation, are presented in Table 1. There were no differences in patient characteristics between groups D and S.

**Table 1:** Patient characteristics, co-morbidity, and data on anesthesia induction and endotracheal intubation

	Group	D (*n* = 182)	S (*n* = 173)	*P*
Sex	F	73 (40.1)	70 (40.5)	0.9460
	M	109 (59.9)	103 (59.5)	
Age (year)		43.0 [30.0;51.0]	41.0 [32.0;54.0]	0.324
Height (cm)		168.1 ± 8.6	166.8 ± 9.8	0.1935
Weight (kg)		67.6 ± 11.6	66.2 ± 12.1	0.2637
Co-morbidity				
Hypertension		19 (10.4)	14 (8.1)	0.4465
Diabetes		3 (1.6)	6 (3.5)	0.3267
Thyroid Disease		1 (0.5)	3 (1.7)	0.3606
Induction and intubation				
Propofol Dose (mg)		107.8 ± 16.2	105.9 ± 16.2	0.2749
Endotracheal intubation				
Method	Laryngoscopy	98 (53.8)	105 (60.7)	0.1925
	Light wand	84 (46.2)	68 (39.3)	0.1925
Number of trials	1	159 (87.4)	148 (85.5)	0.6175
	2	23 (12.6)	25 (14.5)	0.6175

Data are represented as number (percentage), mean ± standard deviation, or median [interquartile range], as appropriate. D, desflurane; S, Sevoflurane

Data on rescue medications for hemodynamic derangements during the anesthesia induction and endotracheal intubation are presented in Supplementary Table 1. As the initial regimen for hypertension or tachycardia, a bolus dose of remifentanil was more frequently administered in group D (*P* < 0.001), and so was a bolus dose of esmolol as the secondary regimen in group D (*P* = 0.0001). The frequency of rescue medications for hypotension did not significantly differ between groups D and S throughout the observation period.

### Comparison between groups D and S using LMM

**Fig. 2:**
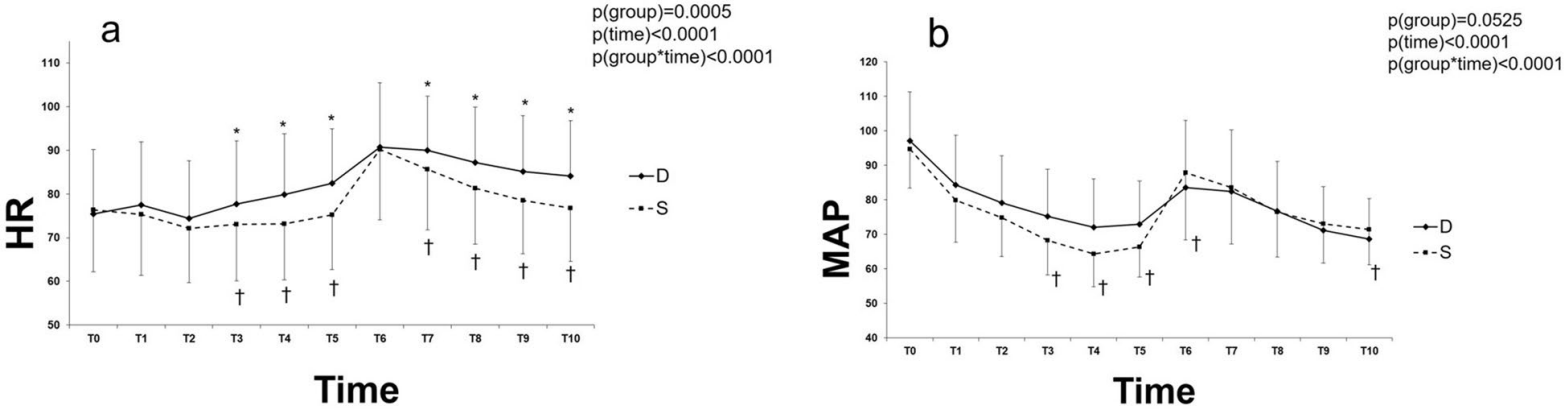
Changes in hemodynamic parameters after anesthesia induction and endotracheal intubation in groups D and S. Data are represented as mean ± standard. D: Desflurane; S: Sevoflurane; a) HR: heart rate; b) MAP: mean arterial blood pressure; T0: baseline; T1-5: 1–5 min after anesthesia induction; T6-10: 1–5 min after endotracheal intubation; *P* group x time: *P* values of group and time interaction obtained by the linear mixed model. ^*^P < 0.05 between groups D and S, ^†^*P* < 0.05 versus T0 between groups D and S

There was no difference in baseline values of HR and MAP between groups D and S. The changes in hemodynamic parameters are presented in Fig. 2. The change in HR over time was significantly different between groups D and S (*P* < 0.0001). In the post hoc analysis, the HR was significantly higher in group D than in group S at T3-10, except at T6 (*P* < 0.05). In group D, the HR was elevated from baseline value at T4-10 (*P* < 0.05), but in group S, initially reduced at T2-3, then elevated at T6-8 (*P* < 0.05). The change in HR from baseline value was significantly different between groups D and S at T3-10, except at T6 (*P* < 0.05) in the post hoc tests. The change in MAP over time was also significantly different between groups D and S (*P* < 0.0001). In the post hoc analysis, the MAP was significantly lower in group S than in group D at T1-5 (*P* < 0.05). The MAP was reduced from baseline value in both groups throughout the study period (*P* < 0.0001). The change in MAP from baseline value was significantly different between groups D and S at T3-6 and T10 (*P* < 0.05) in the post hoc tests. Numeric data of hemodynamic parameters in groups D and S after anesthesia induction and endotracheal intubation are summarized in Supplementary Table 2.

### Comparison between the coeval subgroups using LMM

**Fig. 3:**
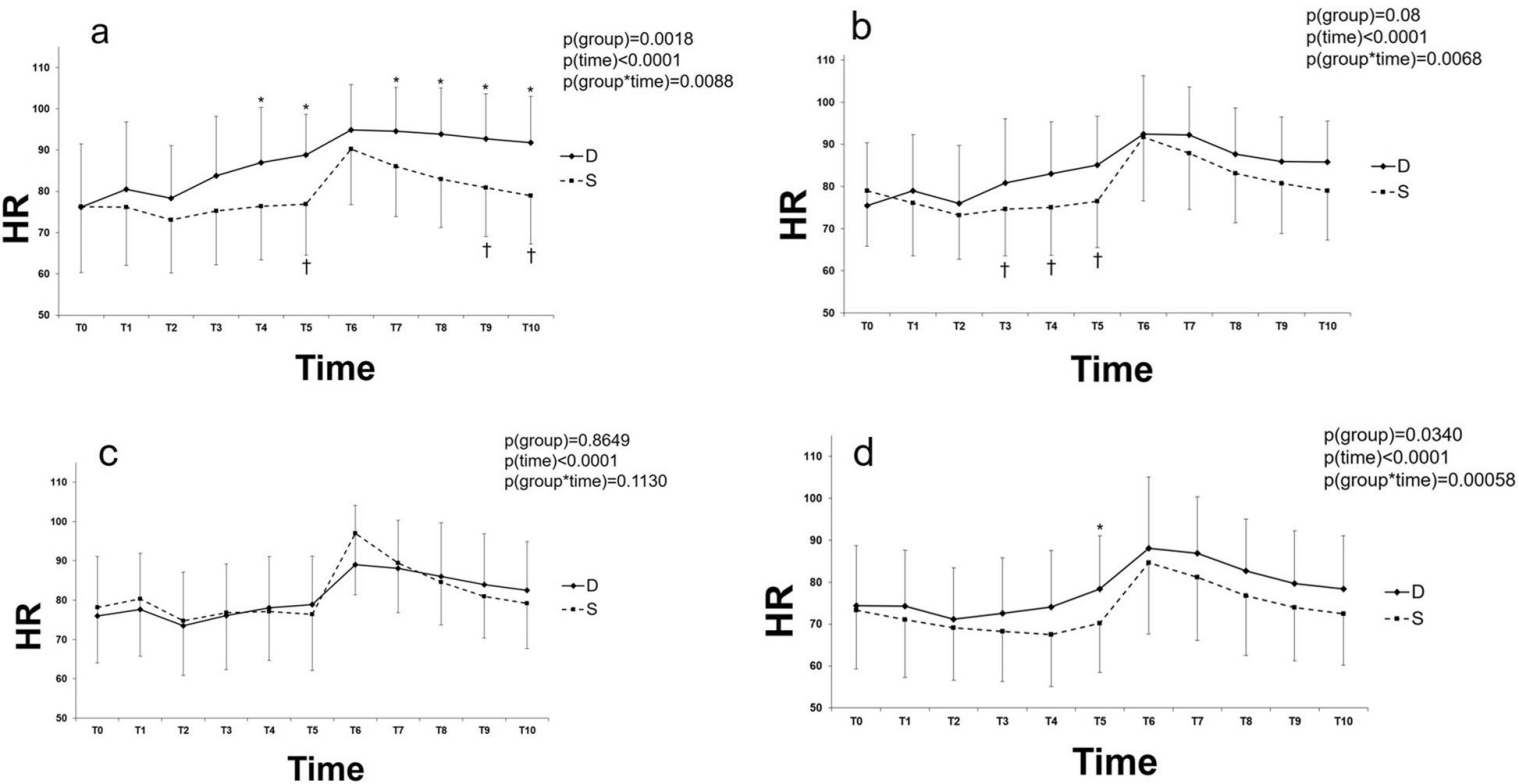
Changes in heart rate (HR) after anesthesia induction and endotracheal intubation in groups D and S. Data are represented as mean ± standard. D: Desflurane; S: Sevoflurane; Patients a) 20–29, b) 30–39, c) 40–49 and d) 50–59 years old; T0: baseline; T1-5: 1–5 min after anesthesia induction; T6-10: 1–5 min after endotracheal intubation; *P* group x time: *P* values of group and time interaction obtained by the linear mixed model. ^*^P < 0.05 between groups D and S, ^†^*P* < 0.05 versus T0 between groups D and S

There were no differences in baseline values of HR and MAP between any coeval subgroup pairs in groups D and S. The changes in HR in the subgroup analysis are shown in Fig. 3. The changes in HR over time were significantly different between all coeval subgroup pairs of groups D and S, except for patients in their 40 s (*P* < 0.01). In the post hoc analysis, the HR was significantly higher in group D than in group S at T4-10, except at T6 (all *P* < 0.05), T4-5 (*P* < 0.05) and T5 (*P* < 0.05) in patients in their 20 s, 30 s and 50 s, respectively. The HR was shown to be significantly elevated from baseline values in subgroups D20 and D30 at T3-10 and T4-10, respectively (*P* < 0.05). Patients in subgroups D40 and D50 had elevated HR from baseline values at T6-9 (*P* < 0.05) and T6-8 (*P* < 0.01), respectively. In group S, the HR was elevated from baseline value at T6-7 in S20, S30, S40, and S50 (*P* < 0.05), whereas the HR was significantly reduced from baseline value at T2 and T3-4 in S30 and S50, respectively (*P* < 0.05). The post hoc tests revealed that the changes in HR from baseline values were significantly different between the coeval subgroup pairs of patients in their 20 s and 30 s at T5 and T9-10 (*P* < 0.05), and T3-5 (*P* < 0.01), respectively. In contrast, no significant difference was found between the coeval subgroup pairs of patients in their 40 s and 50 s.

**Fig. 4:**
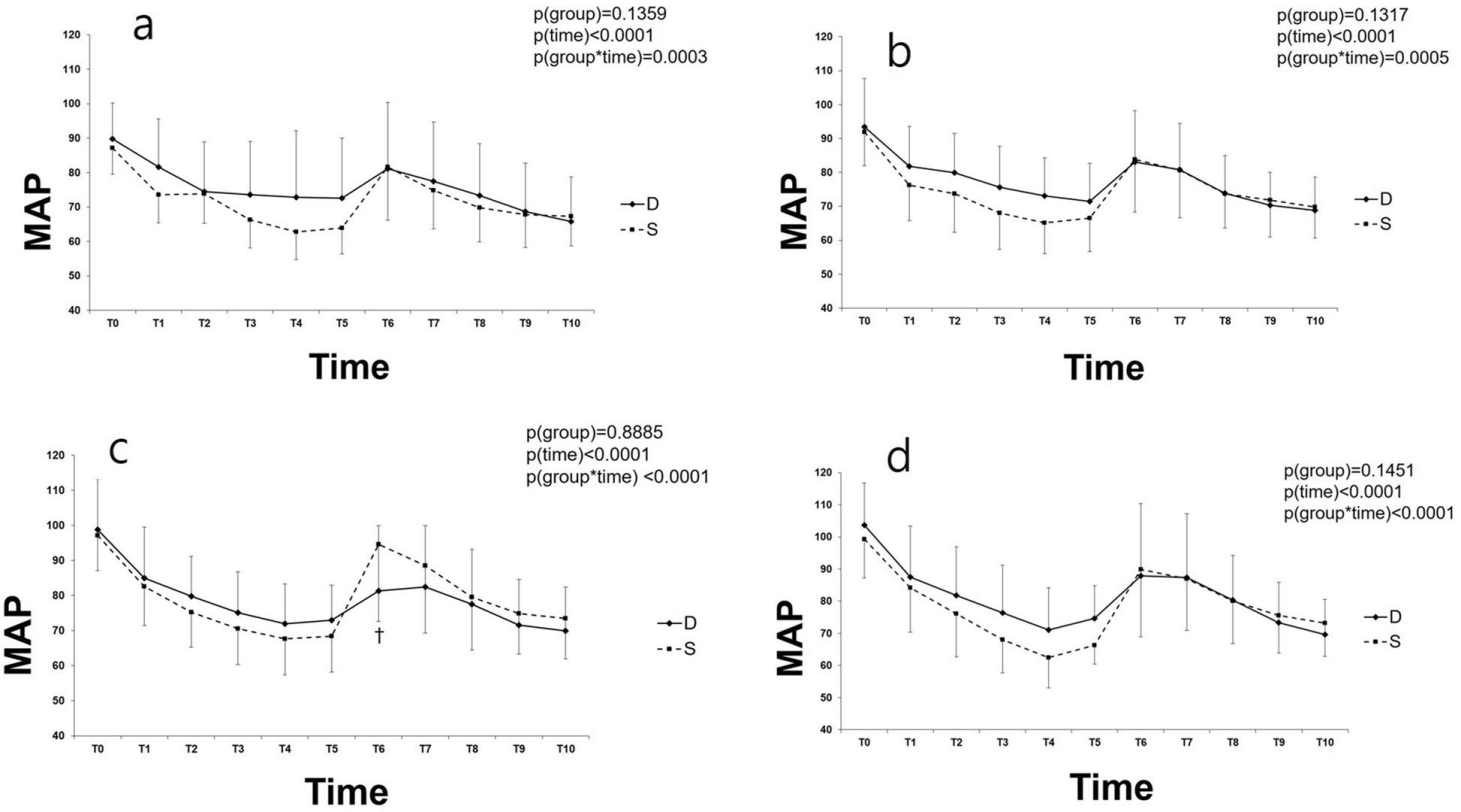
Changes in mean arterial pressure (MAP) after anesthesia induction and endotracheal intubation in groups D and S. Data are represented as mean ± standard. D: Desflurane; S: Sevoflurane; Patients a) 20–29, b) 30–39, c) 40–49 and d) 50–59 years old; T0: baseline; T1-5: 1–5 min after anesthesia induction; T6-10: 1–5 min after endotracheal intubation; *P* group x time: *P* values of group and time interaction obtained by the linear mixed model. ^*^P < 0.05 between groups D and S, ^†^*P* < 0.05 versus T0 between groups D and S

The changes in MAP in the subgroup analysis are shown in Fig. 4. The changes in MAP over time were significantly different between all coeval subgroup pairs of groups D and S (all *P* < 0.001). In the post hoc analysis, the MAP was not shown to be significantly different between subgroups D20 and S20. The MAP was significantly higher in subgroups D30 and D50 than in subgroups S30 and S50 at T3-5 (*P* < 0.05 and *P* < 0.01, respectively). In contrast, the MAP was significantly lower in subgroup D40 than in subgroup S40 at T6 (*P* < 0.05). The MAP was shown to be significantly reduced from baseline values in all coeval subgroups of groups D and S (*P* < 0.05). In the post hoc analysis, however, no significant difference was found between all coeval subgroup pairs of groups D and S. Supplementary Tables 3–6 represent numeric data of hemodynamic parameters after anesthesia induction and endotracheal intubation in age subgroups of groups D and S.

## Discussion

The heart rate was shown to be elevated over time with 1 MAC desflurane, which contrasted to the initial reductions in heart rate with 1 MAC sevoflurane. Furthermore, the heart rate was demonstrated to be higher with desflurane at nearly every time points during the study period compared to that with sevoflurane. In the age-based subgroup analyses, however, significant changes in HR over time were observed only in younger patients in their 20 s and 30 s. The MAP was reduced from baseline value, irrespective of inhalation agent and age.

Although the exact mechanism remains controversial, desflurane has been shown to increase HR, similar to other halogenated anesthetic agents [10]. Ebert et al. demonstrated in a volunteer observational investigation that a rapid increase in desflurane concentration induced sympathetic stimulation, resulting in tachycardia and hypertension [3]. Weiskopf et al. later suggested that desflurane might affect rapid-adapting irritant receptors on the tracheobronchial tree, thereby exerting a sympathetic stimulation effect [11, 12]. Parasympathetic inhibition was recently suggested as a mechanism of HR elevation with desflurane in animal investigations, which employed spectral analysis of HR variability [5, 6].

In those investigations on HR variability, elevations in HR were also shown with increased concentrations of desflurane; however, elevations in MAP were not demonstrated even though desflurane Fi was elevated up to 2 MAC. If the HR was elevated with desflurane *sympathetically*, the MAP should also have been elevated. In this investigation, however, MAP was not elevated with increased Fi of desflurane, which is consistent with animal investigations of parasympathetic inhibition. If the previously suggested irritant receptors are hypothetically present in the human tracheobronchial tree [11–13], the relatively lower Fi of desflurane, employed in this investigation, would not be sufficient to saturate those receptors. In fact, elevation in arterial blood pressure in the previous investigation on sympathetic stimulation was transiently shown with rapid increase in desflurane concentration and reduced soon thereafter [3].

In a previous investigation, HR was shown to be elevated even at < 1 MAC of desflurane, whereas MAP and SVR were shown to be significantly reduced from baseline values, and further elevated as anesthesia deepened, showing an intact direct vascular depressive effect of desflurane at relatively lower concentration [1]. In this investigation, the HR was gradually elevated with increasing desflurane concentration, which contrasts with the immediate reduction in MAP. Therefore, elevations in HR accompanying reductions in MAP could be attributed to the baroreceptor reflex. Although inhalation anesthetic agents are commonly shown to depress baroreceptor reflex activity [14], sevoflurane was demonstrated to attenuate baroreceptor reflex to a greater degree than desflurane at equipotent doses [15]. The ratio of F_ET_/F_i_ of inhalation anesthetics was not significantly different between groups D and S in this investigation (data not shown). Thus, the HR was significantly higher with desflurane than with sevoflurane in response to reductions in arterial blood pressure. Advancing age reportedly blunted the baroreceptor reflex in a previous investigation [16], and thus the magnitude of elevation in HR was shown to be age-related in this investigation.

Desflurane has been avoided during anesthesia induction due to its detrimental hemodynamic effects, especially in patients at risk of ischemic heart diseases which is conventionally thought to increase with advancing age. In this investigation, however, the detrimental hemodynamic responses, previously demonstrated with rapid increases in inspired concentration of desflurane [1–4], were not shown with 1 MAC desflurane, especially in older patients. Moreover, desflurane is supposed to make the arterial blood pressure responses more stable than sevoflurane, preventing an excessive decline in MAP during anesthesia induction and making relatively fewer increases in MAP with endotracheal intubation. Although the HR was significantly higher in group D, a comparison between the coeval subgroups demonstrated significant difference only in younger subgroups. Therefore, it would be prudent not to avoid using desflurane during anesthesia induction in older patients with continuous infusion of remifentanil, especially for brief procedures in which deep anesthesia and rapid emergence are required. In fact, sympathetic stimulation was not observed with high concentration of desflurane [17], and not shown to heighten the cardiac risk when observed [18].

There were several limitations to this investigation. First, no method was employed to explore the mechanism of HR elevation with desflurane, such as measuring sympathetic outflow in the skeletal muscle or kidneys [1–4], or HR variability with spectral analysis [5, 6]. The aim of this investigation was, however, to observe clinical pictures of hemodynamic parameters in ordinary clinical circumstances, maximally simulating standard methods for induction of anesthesia. Therefore, advanced or highly invasive methods have not been employed. In this context, focusing on the procedures of anesthesia induction and endotracheal intubation prevented to explore the clinical relevance of different hemodynamic responses to desflurane and sevoflurane (i.e., the effect on postoperative length of stay). Second, despite a thorough preoperative evaluation, more patients than expected were excluded from the final analysis due to unpredictable airway abnormalities, which could weaken the statistical power of this investigation. Even though the F_ET_ of inhalation anesthetics were elaborately maintained at approximately 80% of the Fi, and mask ventilation was performed in a skillful manner, it was inevitable to encounter inter-practitioner variations in skills of airway maintenance and manual ventilation. It was, however, essential for blindness to assign practitioners randomly and as mentioned earlier, the ratio of F_ET_/Fi of inhalation anesthetics was similar between groups D and S.

In conclusion, inhalation of 1 MAC desflurane during anesthesia induction with propofol bolus and remifentanil continuous infusion at 0.1 μg/kg/min, and endotracheal intubation was more likely to induce elevations in HR than 1 MAC sevoflurane, especially in younger patients. Arterial blood pressure measurements were shown to decrease similarly in groups D and S but maintained greater stability with desflurane. Further investigation is needed to determine the exact mechanism and specific treatment for HR elevation with desflurane.

## Supplementary Information

**Additional file 1.**

## Data Availability

All data generated and/or analyzed during this investigation are included in its supplementary information file.

## Abbreviations

MAC: Minimal alveolar concentration
HR: Heart rate
MAP: Mean arterial blood pressure
F_ET_: End-tidal fraction
Fi: Inspiratory fraction
E_T_CO_2_: End-tidal CO_2_ concentration
LMM: Linear mixed-effect model
