# Supplementary material for: Hemodynamic responses to 1 MAC desflurane inhalation during anesthesia induction with propofol bolus and remifentanil continuous infusion: a prospective randomized single-blind clinical investigation
Source: BMC Anesthesiol. 2023 Feb 22;23:59. doi: 10.1186/s12871-023-02002-6 (PMC9945592; doi:10.1186/s12871-023-02002-6)
Supplement: Supplementary file 1 — Additional file 1. [file 12871_2023_2002_MOESM1_ESM.docx]

Supplementary Table 1. Data on rescue medications to mitigate hemodynamic derangements during anesthesia induction and endotracheal intubation.

|  | Group | | D  (n=182) | S  (n=173) | *P* |
| --- | --- | --- | --- | --- | --- |
| Hypertension or tachycardia | | |  |  |  |
| Rescue medication | | Remifentanil bolus | 101  (55.5%) | 53  (30.6%) | < 0.001 |
|  |  | Esmolol | 16  (8.8%) | 0  (0.0%) | 0.0001 |
| Hypotension | | | | | |
| Anesthetic dose reduction | | Remifentanil | 73  (40.1%) | 68  (39.3%) | 0.8771 |
|  |  | Inhalation agent | 9  (4.9%) | 12  (6.9%) | 0.4266 |
| Rescue medication | | Ephedrine | 4  (2.2%) | 2  (1.2%) | 0.6854 |
|  |  | Phenylephrine | 1  (0.5%) | 0  (0.0%) | > 0.9999 |

Data are represented as number (percentage) of patients. D, Desflurane; S, Sevoflurane.

Supplementary Table 2. Hemodynamic measurements after anesthesia induction and endotracheal intubation in groups D and S.

| Variable | Group | *p*^2^ |  | | After anesthesia induction | | | | | | After endotracheal intubation | | | | | *p*^1^ | | |
| --- | --- | --- | --- | --- | --- | --- | --- | --- | --- | --- | --- | --- | --- | --- | --- | --- | --- | --- |
|  |  |  | T0 | T1 | | T2 | T3 | T4 | T5 | T6 | | T7 | T8 | T9 | T10 | *p*(group) | *p*(time) | *p*(group×time) |
| HR | D |  | 75.4 ± 14.8 | 77.5 ± 14.5 | | 74.4 ± 13.2 | 77.7 ± 14.5 | 79.9 ± 13.9 | 82.5 ± 12.4 | 90.7 ± 14.8 | | 90 ± 12.4 | 87.2 ± 12.8 | 85.1 ± 12.8 | 84.1 ± 12.7 | 0.0005 | <0.0001 | <0.0001 |
|  |  | *p*(time) vs.T0 |  | 0.1579 | | >0.9999 | 0.5262 | 0.0004 | <0.0001 | <0.0001 | | <0.0001 | <0.0001 | <0.0001 | <0.0001 |  |  |  |
|  | S |  | 76.4 ± 14.2 | 75.4 ± 14 | | 72.1 ± 12.5 | 73.1 ± 12.9 | 73.2 ± 12.8 | 75.2 ± 12.5 | 90.2 ± 16.2 | | 85.7 ± 13.9 | 81.3 ± 12.7 | 78.5 ± 12.2 | 76.8 ± 12.2 |  |  |  |
|  |  | *p*(time) vs.T0 |  | >0.9999 | | <0.0001 | 0.014 | 0.075 | 0.285 | <0.0001 | | <0.0001 | 0.0005 | >0.9999 | >0.9999 |  |  |  |
|  |  | *p*(group)  D vs. S | >0.9999 | >0.9999 | | >0.9999 | 0.0163 | <0.0001 | <0.0001 | >0.9999 | | 0.019 | 0.0002 | <0.0001 | <0.0001 |  |  |  |
|  |  | *p*(group*time) vs.T0 |  | 0.098 | | 0.2232 | 0.0006 | <0.0001 | <0.0001 | >0.9999 | | 0.023 | 0.0005 | <0.0001 | <0.0001 |  |  |  |
| MAP | D |  | 97.1 ± 14.2 | 84.3 ± 14.4 | | 79.1 ± 13.6 | 75.2 ± 13.6 | 72.1 ± 14 | 73 ± 12.5 | 83.6 ± 19.5 | | 82.4 ± 17.8 | 76.7 ± 14.4 | 71.2 ± 12.6 | 68.6 ± 11.7 | 0.0525 | <0.0001 | <0.0001 |
|  |  | *p*(time) vs.T0 |  | <0.0001 | | <0.0001 | <0.0001 | <0.0001 | <0.0001 | <0.0001 | | <0.0001 | <0.0001 | <0.0001 | <0.0001 |  |  |  |
|  | S |  | 94.7 ± 11.2 | 79.9 ± 12.2 | | 74.9 ± 11.3 | 68.3 ± 10.1 | 64.4 ± 9.5 | 66.3 ± 8.7 | 87.8 ± 19.4 | | 83.5 ± 16.3 | 76.5 ± 13 | 73 ± 11.4 | 71.4 ± 10.2 |  |  |  |
|  |  | *p*(time) vs.T0 |  | <0.0001 | | <0.0001 | <0.0001 | <0.0001 | <0.0001 | <0.0001 | | <0.0001 | <0.0001 | <0.0001 | <0.0001 |  |  |  |
|  |  | *p*(group)  D vs. S | 0.9243 | 0.0194 | | 0.0162 | <0.0001 | <0.0001 | <0.0001 | 0.3785 | | >0.9999 | >0.9999 | >0.9999 | 0.277 |  |  |  |
|  |  | *p*(group*time) vs.T0 |  | >0.9999 | | >0.9999 | 0.0283 | 0.0059 | 0.0108 | 0.0209 | | >0.9999 | >0.9999 | 0.0907 | 0.0073 |  |  |  |

Data are represented as mean ± standard deviation. D: Desflurane; S: Sevoflurane; HR: heart rate; MAP: mean arterial blood pressure; T0: baseline; T1-5: 1-5 min after anesthesia induction; T6-10: 1-5 min after endotracheal intubation. ^1^: statistical significances calculated by a linear mixed-effect model for repeatedly measured data; *p*(group): between groups D and S; *p*(time): between time points; *p*(group×time): interaction term. ^2^: statistical significances calculated by *post hoc* test using Bonferroni’s correction; *p*(time) vs. T0: comparison to baseline value (T0); *p*(group) D vs. S: comparison between groups D and S; *p*(group*time) vs. T0: comparison to baseline value (T0).

Supplementary Table 3. Hemodynamic measurements after anesthesia induction and endotracheal intubation in subgroups D20 and S20.

| Variable | Subgroup | *P*^2^ |  | After anesthesia induction | | | | | After endotracheal intubation | | | | | | *P*^1^ | | | |
| --- | --- | --- | --- | --- | --- | --- | --- | --- | --- | --- | --- | --- | --- | --- | --- | --- | --- | --- |
|  |  |  | T0 | T1 | T2 | T3 | T4 | T5 | T6 | T7 | T8 | T9 | T10 | *p(*group) | | *p(*time) | *p(*group*time) |  |
| HR | D20 |  | 76.2 ± 15.3] | 80.4 ± 16.3 | 78.3 ± 12.8 | 83.8 ± 14.4 | 87 ± 13.4 | 88.8 ± 9.9 | 94.8 ± 11 | 94.6 ± 10.6 | 93.8 ± 11.2 | 92.7 ± 10.9 | 91.8 ± 11.2 | 0.0018 | | <0.0001 | 0.0088 |  |
|  |  | *p(*time) vs.T0 |  | 0.3297 | >0.9999 | 0.0028 | 0.0001 | <0.0001 | <0.0001 | <0.0001 | <0.0001 | <0.0001 | <0.0001 |  |  |  |  |  |
|  | S20 |  | 76.3 ± 15.9] | 76.2 ± 14.2 | 73.1 ± 12.8 | 75.3 ± 13.1 | 76.3 ± 12.9 | 76.9 ± 12.4 | 90.2 ± 13.4 | 86.1 ± 12.2 | 82.9 ± 11.7 | 80.9 ± 11.8 | 79 ± 11.8 |  |  |  |  |  |
|  |  | *p(*time) vs.T0 |  | >0.9999 | >0.9999 | >0.9999 | >0.9999 | >0.9999 | 0.0001 | 0.0048 | 0.4689 | >0.9999 | >0.9999 |  |  |  |  |  |
|  |  | *p(*group)  D20 vs. S20 | >0.9999 | >0.9999 | >0.9999 | 0.1308 | 0.0128 | 0.0008 | >0.9999 | 0.0239 | 0.0017 | 0.0004 | 0.0001 |  | |  |  |  |
|  |  | *p(*group*time) vs.T0 |  | >0.9999 | >0.9999 | 0.1406 | 0.0971 | 0.0264 | >0.9999 | 0.3515 | 0.057 | 0.0174 | 0.0053 |  | |  |  |  |
| MAP | D20 |  | 89.7 ± 10.4] | 81.6 ± 13.9 | 74.4 ± 14.5 | 73.6 ± 15.4 | 72.8 ± 19.3 | 72.5 ± 17.6 | 81.1 ± 19.2 | 77.4 ± 17.3 | 73.3 ± 15.1 | 68.6 ± 14.1 | 65.7 ± 12.9 | 0.1359 | | <0.0001 | 0.0003 |  |
|  |  | *p(*time) vs.T0 |  | <0.0001 | <0.0001 | <0.0001 | <0.0001 | <0.0001 | 0.0214 | <0.0001 | <0.0001 | <0.0001 | <0.0001 |  |  |  |  |  |
|  | S20 |  | 87.1 ± 7.6] | 73.6 ± 8.2 | 73.8 ± 8.6 | 66.3 ± 8.2 | 62.8 ± 8.1 | 63.8 ± 7.4 | 81.6 ± 15.5 | 74.8 ± 11.2 | 69.8 ± 9.9 | 67.8 ± 9.5 | 67.3 ± 8.5 |  |  |  |  |  |
|  |  | *p(*time) vs.T0 |  | <0.0001 | <0.0001 | <0.0001 | <0.0001 | <0.0001 | >0.9999 | <0.0001 | <0.0001 | <0.0001 | <0.0001 |  |  |  |  |  |
|  |  | *p(*group)  D20 vs. S20 | >0.9999 | 0.0713 | >0.9999 | 0.2371 | 0.1062 | 0.172 | >0.9999 | >0.9999 | >0.9999 | >0.9999 | >0.9999 |  | |  |  |  |
|  |  | *p(*group*time) vs.T0 |  | 0.3575 | >0.9999 | >0.9999 | >0.9999 | >0.9999 | >0.9999 | >0.9999 | >0.9999 | >0.9999 | >0.9999 |  | |  |  |  |

Data are represented as mean ± standard deviation. D20: Desflurane in age of 20-29 years; S20: Sevoflurane in age of 20-29 years; HR: heart rate; MAP: mean arterial blood pressure; T0: baseline; T1-5: 1-5 min after anesthesia induction; T6-10: 1-5 min after endotracheal intubation. ^1^: statistical significances calculated by a linear mixed-effect model for repeatedly measured data; *p*(group): between groups D and S; *p*(time): between time points; *p*(group×time): interaction term. ^2^: statistical significances calculated by *post hoc* test using Bonferroni’s correction; *p*(time) vs. T0: comparison to baseline value (T0); *p*(group) D vs. S: comparison between groups D and S; *p*(group*time) vs. T0: comparison to baseline value (T0).

Supplementary Table 4. Hemodynamic measurements after anesthesia induction and endotracheal intubation in subgroups D30 and S30.

| Variable | Subgroup | *P*^2^ |  | After anesthesia induction | | | | | After endotracheal intubation | | | | | *P*^1^ | | |
| --- | --- | --- | --- | --- | --- | --- | --- | --- | --- | --- | --- | --- | --- | --- | --- | --- |
|  |  |  | T0 | T1 | T2 | T3 | T4 | T5 | T6 | T7 | T8 | T9 | T10 | *p(*group) | *p(*time) | *p(*group*time) |
| HR | D30 |  | 75.5 ± 14.8] | 79 ± 13.4 | 76 ± 13.7 | 80.8 ± 15.2 | 83 ± 12.3 | 85.1 ± 11.7 | 92.4 ± 13.9 | 92.2 ± 11.5 | 87.7 ± 10.9 | 85.9 ± 10.5 | 85.9 ± 9.6 | 0.08 | <0.0001 | 0.0068 |
|  |  | *p(*time) vs.T0 |  | >0.9999 | >0.9999 | 0.2586 | 0.0254 | 0.0068 | <0.0001 | <0.0001 | 0.0001 | 0.0009 | 0.0027 |  |  |  |
|  | S30 |  | 79 ± 13.2] | 76.1 ± 12.5 | 73.2 ± 10.4 | 74.7 ± 11.1 | 75 ± 11.4 | 76.5 ± 10.9 | 91.7 ± 15.1 | 87.9 ± 13.4 | 83.1 ± 11.7 | 80.7 ± 11.9 | 79 ± 11.8 |  |  |  |
|  |  | *p(*time) vs.T0 |  | >0.9999 | 0.0146 | 0.449 | >0.9999 | >0.9999 | <0.0001 | 0.0031 | >0.9999 | >0.9999 | >0.9999 |  |  |  |
|  |  | *p(*group)  D30 vs. S30 | >0.9999 | >0.9999 | >0.9999 | 0.4149 | 0.0438 | 0.0388 | >0.9999 | >0.9999 | 0.7851 | 0.4227 | 0.1485 |  |  |  |
|  |  | *p(*group*time) vs.T0 |  | 0.3511 | 0.404 | 0.0085 | 0.0048 | 0.0057 | >0.9999 | 0.8742 | 0.5639 | 0.2485 | 0.0883 |  |  |  |
| MAP | D30 |  | 93.4 ± 14.3] | 81.8 ± 11.8 | 79.9 ± 11.7 | 75.7 ± 12.1 | 73.1 ± 11.2 | 71.4 ± 11.3 | 83.1 ± 15.1 | 80.7 ± 13.7 | 73.9 ± 11.1 | 70.3 ± 9.7 | 68.8 ± 9.8 | 0.1317 | <0.0001 | 0.0005 |
|  |  | *p(*time) vs.T0 |  | <0.0001 | <0.0001 | <0.0001 | <0.0001 | <0.0001 | 0.0078 | <0.0001 | <0.0001 | <0.0001 | <0.0001 |  |  |  |
|  | S30 |  | 91.8 ± 10] | 76.2 ± 10.5 | 73.7 ± 11.4 | 68 ± 10.7 | 65.1 ± 9.1 | 66.5 ± 9.8 | 83.8 ± 15.5 | 80.7 ± 14 | 73.7 ± 10.1 | 71.8 ± 10.9 | 69.8 ± 9.1 |  |  |  |
|  |  | *p(*time) vs.T0 |  | <0.0001 | <0.0001 | <0.0001 | <0.0001 | <0.0001 | 0.0344 | <0.0001 | <0.0001 | <0.0001 | <0.0001 |  |  |  |
|  |  | *p(*group)  D30 vs. S30 | >0.9999 | 0.3108 | 0.2108 | 0.037 | 0.0041 | 0.0165 | >0.9999 | >0.9999 | >0.9999 | >0.9999 | >0.9999 |  |  |  |
|  |  | *p(*group*time) vs.T0 |  | >0.9999 | >0.9999 | 0.6508 | 0.1864 | 0.6088 | >0.9999 | >0.9999 | >0.9999 | >0.9999 | >0.9999 |  |  |  |

Data are represented as mean ± standard deviation. D30: Desflurane in age of 30-39 years; S30: Sevoflurane in age of 30-39 years; HR: heart rate; MAP: mean arterial blood pressure; T0: baseline; T1-5: 1-5 min after anesthesia induction; T6-10: 1-5 min after endotracheal intubation. ^1^: statistical significances calculated by a linear mixed-effect model for repeatedly measured data; *p*(group), between groups D and S; *p*(time): between time points; *p*(group×time): interaction term. ^2^: statistical significances calculated by *post hoc* test using Bonferroni’s correction; *p*(time) vs. T0: comparison to baseline value (T0); *p*(group) D vs. S: comparison between groups D and S; *p*(group*time) vs. T0: comparison to baseline value (T0).

| Variable | Subgroup | p^2^ |  | After anesthesia induction | | | | | After endotracheal intubation | | | | | p^1^ | | |
| --- | --- | --- | --- | --- | --- | --- | --- | --- | --- | --- | --- | --- | --- | --- | --- | --- |
|  |  |  | T0 | T1 | T2 | T3 | T4 | T5 | T6 | T7 | T8 | T9 | T10 | *p(*group) | *p(*time) | *p(*group*time) |
| HR | D40 |  | 75.9 ± 15.1] | 77.6 ± 14.3 | 73.5 ± 13.6 | 76.1 ± 13.2 | 78.1 ± 13 | 78.8 ± 12.3 | 89 ± 15 | 88 ± 12.2 | 86 ± 13.7 | 83.9 ± 13 | 82.5 ± 12.5 | 0.8649 | <0.0001 | 0.113 |
|  |  | *p(*time) vs.T0 |  | >0.9999 | >0.9999 | >0.9999 | >0.9999 | >0.9999 | <0.0001 | <0.0001 | 0.0027 | 0.0356 | 0.2675 |  |  |  |
|  | S40 |  | 78.2 ± 14] | 80.4 ± 14.6 | 74.7 ± 13.8 | 76.8 ± 14.4 | 77.1 ± 12.4 | 76.4 ± 14.3 | 97 ± 15.7 | 89.4 ± 12.6 | 84.6 ± 10.8 | 81 ± 10.6 | 79.2 ± 11.5 |  |  |  |
|  |  | *p(*time) vs.T0 |  | >0.9999 | >0.9999 | >0.9999 | >0.9999 | >0.9999 | <0.0001 | 0.0014 | 0.9104 | >0.9999 | >0.9999 |  |  |  |
|  |  | *p(*group)  D40 vs. S40 | >0.9999 | >0.9999 | >0.9999 | >0.9999 | >0.9999 | >0.9999 | 0.1965 | >0.9999 | >0.9999 | >0.9999 | >0.9999 |  |  |  |
|  |  | *p(*group*time) vs.T0 |  | >0.9999 | >0.9999 | >0.9999 | >0.9999 | >0.9999 | >0.9999 | >0.9999 | >0.9999 | >0.9999 | >0.9999 |  |  |  |
| MAP | D40 |  | 98.8 ± 14.5] | 85 ± 14.5 | 79.8 ± 11.4 | 75.1 ± 11.7 | 72 ± 11.4 | 72.9 ± 10 | 81.4 ± 18.6 | 82.5 ± 17.4 | 77.6 ± 15.7 | 71.6 ± 13 | 69.9 ± 12.6 | 0.8885 | <0.0001 | <0.0001 |
|  |  | *p(*time) vs.T0 |  | <0.0001 | <0.0001 | <0.0001 | <0.0001 | <0.0001 | <0.0001 | <0.0001 | <0.0001 | <0.0001 | <0.0001 |  |  |  |
|  | S40 |  | 97.1 ± 10] | 82.6 ± 11.1 | 75.3 ± 10 | 70.5 ± 10.3 | 67.7 ± 10.4 | 68.4 ± 10.2 | 94.6 ± 22 | 88.6 ± 19.3 | 79.6 ± 15.1 | 74.8 ± 11.5 | 73.5 ± 11.5 |  |  |  |
|  |  | *p(*time) vs.T0 |  | <0.0001 | <0.0001 | <0.0001 | <0.0001 | <0.0001 | >0.9999 | 0.1012 | <0.0001 | <0.0001 | <0.0001 |  |  |  |
|  |  | *p(*group)  D40 vs. S40 | >0.9999 | >0.9999 | 0.5916 | 0.662 | 0.4212 | 0.0699 | 0.0238 | >0.9999 | >0.9999 | >0.9999 | >0.9999 |  |  |  |
|  |  | *p(*group*time) vs.T0 |  | >0.9999 | >0.9999 | >0.9999 | >0.9999 | >0.9999 | 0.0087 | >0.9999 | >0.9999 | >0.9999 | >0.9999 |  |  |  |

Supplementary Table 5. Hemodynamic measurements after anesthesia induction and endotracheal intubation in subgroups D40 and S40.

Data are represented as mean ± standard deviation. D40: Desflurane in age of 40-49 years; S40: Sevoflurane in age of 40-49 years; HR: heart rate; MAP: mean arterial blood pressure; T0: baseline; T1-5: 1-5 min after anesthesia induction; T6-10: 1-5 min after endotracheal intubation. ^1^: statistical significances calculated by a linear mixed-effect model for repeatedly measured data; *p*(group), between groups D and S; *p*(time): between time points; *p*(group×time): interaction term. ^2^: statistical significances calculated by *post hoc* test using Bonferroni’s correction; *p*(time) vs. T0: comparison to baseline value (T0); *p*(group) D vs. S: comparison between groups D and S; *p*(group*time) vs. T0: comparison to baseline value (T0).

Supplementary Table 6. Hemodynamic measurements after anesthesia induction and endotracheal intubation in the subgroups D50 and S50.

| Variable | Subgroup | *P*^2^ |  | After anesthesia induction | | | | | After endotracheal intubation | | | | | | *P*^1^ | | |
| --- | --- | --- | --- | --- | --- | --- | --- | --- | --- | --- | --- | --- | --- | --- | --- | --- | --- |
|  |  |  | T0 | T1 | T2 | T3 | T4 | T5 | T6 | T7 | T8 | T9 | T10 | *p(*group) | | *p(*time) | *p(*group*time) |
| HR | D50 |  | 74.4 ± 14.3] | 74.2 ± 13.4 | 71.1 ± 12.4 | 72.5 ± 13.2 | 74 ± 13.4 | 78.3 ± 12.8 | 88 ± 17.1 | 86.9 ± 13.4 | 82.7 ± 12.3 | 79.7 ± 12.6 | 78.4 ± 12.7 | 0.034 | | <0.0001 | 0.0058 |
|  |  | *p(*time) vs.T0 |  | >0.9999 | 0.7138 | >0.9999 | >0.9999 | >0.9999 | <0.0001 | <0.0001 | 0.0002 | 0.1538 | >0.9999 |  |  |  |  |
|  | S50 |  | 73.3 ± 14] | 71 ± 13.7 | 69.1 ± 12.5 | 68.2 ± 11.9 | 67.5 ± 12.5 | 70.2 ± 11.7 | 84.6 ± 17 | 81.1 ± 15 | 76.8 ± 14.2 | 73.9 ± 12.6 | 72.4 ± 12.2 |  |  |  |  |
|  |  | *p(*time) vs.T0 |  | >0.9999 | 0.0854 | 0.0422 | 0.0368 | 0.0659 | <0.0001 | 0.0015 | >0.9999 | >0.9999 | >0.9999 |  |  |  |  |
|  |  | *p(*group)  D50 vs. S50 | >0.9999 | >0.9999 | >0.9999 | 0.7791 | 0.1033 | 0.0118 | >0.9999 | 0.3416 | 0.2257 | 0.182 | 0.1342 |  | |  |  |
|  |  | *p(*group*time) vs.T0 |  | >0.9999 | >0.9999 | >0.9999 | >0.9999 | 0.2257 | >0.9999 | >0.9999 | >0.9999 | >0.9999 | >0.9999 |  | |  |  |
| MAP | D50 |  | 103.6 ± 13.2] | 87.5 ± 15.8 | 81.8 ± 15.1 | 76.4 ± 14.8 | 71.1 ± 13.1 | 74.7 ± 10.1 | 87.9 ± 22.5 | 87.4 ± 19.8 | 80.3 ± 13.9 | 73.3 ± 12.6 | 69.6 ± 11 | 0.1454 | | <0.0001 | <0.0001 |
|  |  | *p(*time) vs.T0 |  | <0.0001 | <0.0001 | <0.0001 | <0.0001 | <0.0001 | <0.0001 | <0.0001 | <0.0001 | <0.0001 | <0.0001 |  |  |  |  |
|  | S50 |  | 99.2 ± 12.1] | 84.2 ± 13.7 | 76.1 ± 13.4 | 68 ± 10.3 | 62.5 ± 9.5 | 66.2 ± 5.8 | 89.8 ± 30.9 | 86.9 ± 16 | 80.1 ± 13.3 | 75.5 ± 11.7 | 73.2 ± 10.4 |  |  |  |  |
|  |  | *p(*time) vs.T0 |  | <0.0001 | <0.0001 | <0.0001 | <0.0001 | <0.0001 | 0.0266 | <0.0001 | <0.0001 | <0.0001 | <0.0001 |  |  |  |  |
|  |  | *p(*group)  D50 vs. S50 | 0.776 | >0.9999 | 0.3976 | 0.007 | 0.001 | 0.0001 | >0.9999 | >0.9999 | >0.9999 | >0.9999 | 0.7681 |  | |  |  |
|  |  | *p(*group*time) vs.T0 |  | >0.9999 | >0.9999 | >0.9999 | >0.9999 | >0.9999 | >0.9999 | >0.9999 | >0.9999 | 0.5279 | 0.1061 |  | |  |  |

Data are represented as mean ± standard deviation. D50: Desflurane in age of 50-59 years; S50: Sevoflurane in age of 50-59 years; HR: heart rate; MAP: mean arterial blood pressure; T0: baseline; T1-5: 1-5 min after anesthesia induction; T6-10: 1-5 min after endotracheal intubation. ^1^: statistical significances calculated by a linear mixed-effect model for repeatedly measured data; *p*(group), between groups D and S; *p*(time): between time points; *p*(group×time): interaction term. ^2^: statistical significances calculated by *post hoc* test using Bonferroni’s correction; *p*(time) vs. T0: comparison to baseline value (T0); *p*(group) D vs. S: comparison between groups D and S; *p*(group*time) vs. T0: comparison to baseline value (T0).
